# The Cortisol Response to Anticipated Intergroup Interactions Predicts Self-Reported Prejudice

**DOI:** 10.1371/journal.pone.0033681

**Published:** 2012-03-19

**Authors:** Erik Bijleveld, Daan Scheepers, Naomi Ellemers

**Affiliations:** 1 Department of Psychology, Utrecht University, Utrecht, The Netherlands; 2 Institute for Psychological Research, Leiden Institute for Brain and Cognition, Leiden University, Leiden, The Netherlands; Cajal Institute, Consejo Superior de Investigaciones Científicas, Spain

## Abstract

**Objectives:**

While prejudice has often been shown to be rooted in experiences of threat, the biological underpinnings of this threat–prejudice association have received less research attention. The present experiment aims to test whether activations of the hypothalamus-pituitary-adrenal (HPA) axis, due to anticipated interactions with out-group members, predict self-reported prejudice. Moreover, we explore potential moderators of this relationship (i.e., interpersonal similarity; subtle vs. blatant prejudice).

**Methodology/Principal findings:**

Participants anticipated an interaction with an out-group member who was similar or dissimilar to the self. To index HPA activation, cortisol responses to this event were measured. Then, subtle and blatant prejudices were measured via questionnaires. Findings indicated that only when people anticipated an interaction with an out-group member who was dissimilar to the self, their cortisol response to this event significantly predicted subtle (*r* = .50) and blatant (*r* = .53) prejudice.

**Conclusions:**

These findings indicate that prejudicial attitudes are linked to HPA-axis activity. Furthermore, when intergroup interactions are interpreted to be about individuals (and not so much about groups), experienced threat (or its biological substrate) is less likely to relate to prejudice. This conclusion is discussed in terms of recent insights from social neuroscience.

## Introduction

People have a deeply-rooted tendency to dislike social or ethnic groups of which they themselves are not a member [1]. Often referred to as *prejudice*, this tendency is considered to be a central cause of various societal problems. Accordingly, researchers have extensively studied these negative attitudes in order to delineate their causes and consequences. An important finding from this field of research is that the experience of group-related threats—regardless of their exact source—boosts prejudicial attitudes and behaviors. Whether threat stems from competition with other groups for scarce resources, from conflicting values, or from potentially being harmed or devaluated by an *out-group*, it non-specifically increases prejudice [2].

As previous research on this topic has mainly relied on self-report measures of threat, the neurophysiological basis of the threat–prejudice relationship has remained rather obscure. In the present article, we propose that the extent to which the hypothalamic–pituitary–adrenal (HPA) axis is activated during intergroup situations is predictive of expressions of prejudice. This proposal is supported by the idea that, although they have been examined in different fields of research, cortisol (i.e., the end-point of the HPA axis) and prejudice have the same antecedents and serve similar functions. That is, both elevations of cortisol and increases in prejudice have been proposed to be caused by social threats. Furthermore, both cortisol and prejudice have been proposed to have restorative functions, in that they help people to (re)gain control over the situation after threats have been encountered. We will now address these ideas in greater detail.

The secretion of cortisol is known to occur in response to stressors that are (a) uncontrollable and (b) involve social evaluation [3]. For example, a procedure that has both of these components is the Trier Social Stress Test, in which people are required to give a speech and perform a cognitive task in front of an evaluating audience [4]. This procedure reliably leads to the secretion of cortisol. Similarly, social-group-related threats also seem to have the potential to cause elevations in cortisol. For example, in a recent study, female subjects interacted with a male confederate who was introduced as endorsing sexist attitudes. Results indicated that subjects who were predisposed towards detecting sexism in society showed elevated cortisol due to this interaction [5]. So, the experience of social threat—e.g., due to inter-group situations that are perceived as highly stressful—can cause cortisol elevations [4]–[7]. Importantly, many studies have shown that out-group threats *also* increase out-group prejudice [2]. So, social threat turns out to be a cause of both cortisol elevations and expressions of prejudice, raising the possibility that prejudice is underpinned by (or at least correlated with) HPA-axis activation.

In addition, elevations of cortisol and expressions of prejudice also overlap in their functionality, in that both have been proposed to have restorative functions. For cortisol, studies have shown that while social stressors normally decrease people's mood, administration of cortisol ameliorates this effect [8]. Similar effects have been found in patients who suffer from post-traumatic stress disorder, who benefit from cortisol administration because this inhibits the retrieval of traumatic memories [9]. These findings support the perspective that cortisol helps people to adaptively regain control over the situation after stressors are experienced, via the modulation of cognitive and metabolic processes [10]–[12]. Intriguingly, similar functions have often been ascribed to prejudice. For example, expressing prejudice has been found to restore people's self-esteem after this has been harmed [13]. Similarly, expressing prejudice towards other groups increases the extent to which people identify with and value their own group, especially after their own group is threatened in any way [14]. Thus, there is also overlap in the functionality of cortisol and prejudice, in that both broadly restore well-being and serve to regain control after threats have been encountered.

The lines of reasoning addressed above indicate that cortisol and prejudice have shared antecedents (i.e., social threat) and compatible functions (i.e., restoring well-being, regaining control). It is thus an interesting possibility that they are intertwined, and that cortisol responses (due to intergroup situations) are predictive of expressions of prejudice. Indeed, some research suggests that HPA functioning is directly related to prejudice-related phenomena. Specifically, one study showed that HPA-axis dysregulation correlates with internalized racism (i.e., the extent to which people endorse negative stereotypes about their own group) [15]. Yet, while this finding is generally in line with the ideas put forward in this paper, this previous study did not address prejudice directed at other groups (i.e., out-groups). Moreover, it is currently less clear whether *acute* cortisol responses to intergroup stressors are linked to prejudice. Accordingly, it is the main purpose of the present experiment to establish whether a relation between acute cortisol elevations and self-reported prejudice towards an out-group exists. In our study, participants first anticipate an intergroup interaction, while their cortisol response to this anticipation is measured. Next, participants engage in a (bogus) inter-group interaction, after which self-report measures of prejudice are administered.

While this procedure allows us to test our main hypothesis, we also aim to explore two boundary conditions of the anticipated cortisol–prejudice relation. First, on the basis of previous research on threats in intergroup situations, we expect that the cortisol–prejudice relation is strongest for forms of prejudice that are difficult to suppress (i.e., subtle rather than blatant forms of prejudice) [16], [17]. Second, the cortisol–prejudice relation is expected be weaker when people feel similar to the out-group member with whom they will interact. That is, in such contexts of interpersonal similarity, people perceive social interactions—and any threats that are accompanied with it—to be about individuals, rather than about groups [18]–[20]. Thus, if people experience threats and/or have elevated cortisol in such a context, these do not necessarily relate to the out-group as a whole. To test this additional idea, half of the participants were led to believe they were interpersonally similar (vs. dissimilar) to the bogus out-group member with whom they are about to interact.

## Methods

### Ethics statement

This study was approved by the Ethics committee of the Leiden University Institute for Psychological Research. All participants gave written informed consent.

### Participants

Forty-one male, Caucasian students participated (mean age = 21.4) in a computerized laboratory experiment. They took part in individual sessions that we planned in the late morning or in the afternoon, and received €6 for their participation. Participants were randomly divided across the similar and dissimilar conditions.

### Procedure

When participants arrived in the laboratory, we took a baseline measure of salivary cortisol using a *salivette*, a cotton swab designed for taking saliva samples. Subsequently, participants were led to believe they were to do a task in cooperation with an out-group member—in this case, a Moroccan-Dutch woman who was actually a confederate. This task, so they were told, constituted a word-search task in which they had to find words in a letter matrix. To make the procedure as credible as possible, participants were shown a picture of their partner for the task: A woman with Moroccan facial features who wore a headscarf, representing a highly stigmatized ethnic minority-group in the Netherlands.

After this introduction, participants filled out a questionnaire, ostensibly to determine their *similarity* to the bogus other participant. These questionnaire items were actually taken from a personality test. Participants received feedback on their responses to these items, depending on the condition to which they were assigned. Participants in the dissimilar condition were informed that they were very dissimilar on a dispositional characteristic (termed ‘search style’), that was supposedly highly relevant to the word-search task at hand. Conversely, participants in the similar condition were told that they were highly similar on this characteristic. As a manipulation check, participants were then asked whether they thought they were the interpersonally similar to or different from their interaction partner. All participants answered this question in line with the information they were provided with, indicating that the manipulation was successful [21]–[23]. Next, participants filled out some filler material, as the human cortisol response is known to take several minutes to unfold [3]. After this delay, participants completed the second measure of salivary cortisol, which was timed such that it tapped the effects of the anticipated interaction.

To further ensure that the procedure was credible to the participants, they completed a word-search task. This task, that was taken from previous experiments on intergroup interactions [24], [25], required participants to search words in a letter matrix. During task performance, participants could see the bogus out-group member (‘Naima’) on a webcam window, raising the suggestion to participants that she was also doing the task at the same time.

Finally, we measured the extent to which participants expressed prejudice using a standard questionnaire [26], which was tailored to the relevant out-group (i.e., Moroccan immigrants in the Netherlands). It measured *subtle prejudice* with seven items (α = .74), such as ‘Moroccans living here teach their children values and skills different from those required to be successful in the Netherlands’. It measured *blatant prejudice* (i.e., a more aggressive, overt form of prejudice), with five items (α = .74), such as ‘Moroccans belong to an inferior race, which explains why they are less successful in society than are most Dutch people’. Participants indicated their agreement to these statements on a 7-point Likert-type scale.

## Results

### Preliminary analyses

Saliva samples were assayed for cortisol by a specialized laboratory. We computed a cortisol elevation score by subtracting baseline cortisol from the second cortisol measure. First, we checked whether this score was significantly different from zero (which would indicate group-level changes in cortisol level), which was not the case, *t*(40) = .05, *p* = .96. In addition, cortisol elevation was not significantly different for participants in the similar vs. the dissimilar condition, *t*(39) = 1.44, *p* = .16. Both observations are not surprising [16], as establishing group-level increases in cortisol requires more intense stressors than the mere anticipation of an intergroup interaction (e.g., the Trier Social Stress Test). Nevertheless, and this was crucial for testing our hypothesis, there was ample between-subjects variance in cortisol elevation (*M* = −.05 nmol/L, *SD* = 7.07). The dataset is included as Supporting Information (Data S1).

### Subtle prejudice

**Figure 1:**
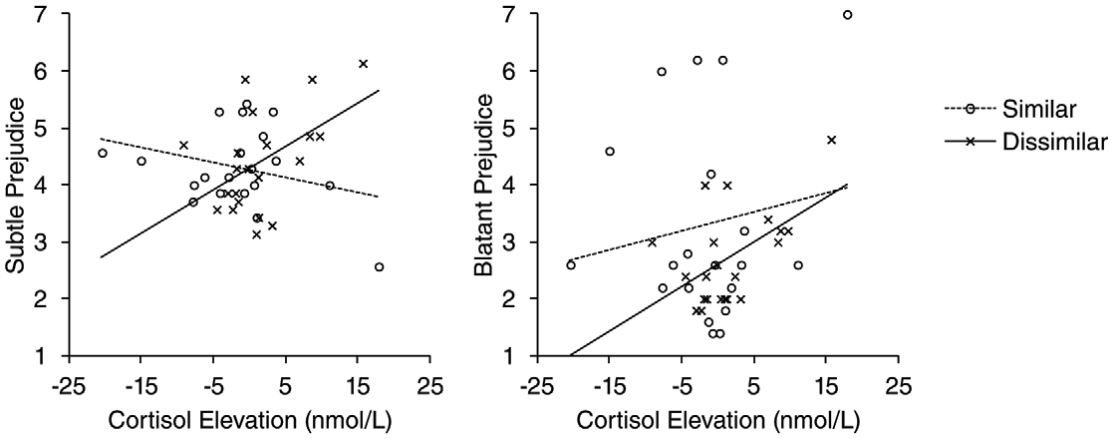
Subtle and blatant prejudice as a function of cortisol elevation. Lines indicate the direction of the relation between cortisol and self-reported prejudice. In both panels, solid lines represent the statistically reliable relation between cortisol (due to an anticipated interaction with an out-group member dissimilar to the self), and subtle and blatant prejudice, respectively.

To test whether subtle prejudice was related to cortisol elevation, and whether this was different for the similar vs. dissimilar conditions, we regressed subtle prejudice on similarity and cortisol elevation (centered). The main effects of these predictors were not significant, *t*'s<.45, *p*'s>.66. Next, the similarity×cortisol interaction was added to the model. This interaction was significant, β = .54, *t* = 2.90, *p* = .006, indicating that the relationship between cortisol and prejudice was different in the similar vs. dissimilar condition. To examine the nature of this interaction, we computed correlations between cortisol and subtle prejudice separately for the similar and the dissimilar conditions. In line with our predictions, this analysis revealed that cortisol elevation predicted subtle prejudice in the dissimilar condition, *r* = .50, *p* = .021, but not in the similar condition, *r* = −.31, *p* = .191 (Figure 1, left).

### Blatant prejudice

The same regression analysis was conducted, now with blatant prejudice as a dependent variable. Neither main effects of cortisol and similarity, nor their interaction, was significant, *t*'s<1.75, *p*'s>.095. Visual inspection of the data (Figure 1, right), however, suggested that our sample included four participants who reported much more blatant prejudice than the others (≥6). When these individuals were excluded from analysis, a similar pattern emerged as for subtle prejudice. That is, the similarity×cortisol interaction was significant, β = .56, *t* = 2.67, *p* = .012. As was the case for subtle prejudice, this interaction indicated that cortisol elevation predicted blatant prejudice in the dissimilar condition, *r* = .53, *p* = .013, but not in the similar condition, *r* = −.28, *p* = .295.

## Discussion

We found that the cortisol response, after people anticipated an intergroup interaction, predicted the amount of self-reported prejudice towards the relevant out-group (Moroccans, in this case). This finding suggests that prejudicial attitudes are, on the biological level, mirrored by HPA-axis activity. This idea fits well with the rationale that exhibiting prejudice may be a way to restore one's own well-being and regain control after social stress has encountered [14], and that cortisol supports this function [11]. We found no differences for subtle and blatant prejudice, suggesting that both forms are affected by cortisol, at least as long as they are expressed via self-reports. Importantly, however, the relation between cortisol and prejudice was moderated by interpersonal similarity. As expected, only when they were due to an anticipated interaction with an out-group member dissimilar to the self, cortisol responses were related to prejudice. This moderation is in line with the idea that when intergroup interactions are interpreted to be about individuals (and not so much about groups), any experienced threats are not relevant to the group as a whole.

It is important to note that the design of the present study does not allow for conclusions about the causal direction of the cortisol–prejudice relationship. In line with this limitation, our theoretical analysis merely suggests that the cortisol response *co-occurs* with prejudice. Still, we can speculate about the causal order of this relationship, and there are indeed several possibilities. First, the HPA-axis response (that occurs initially) may be interpreted as an aversive bodily sensation, which may be attributed to the out-group, thus causing prejudice [27], [28]. Second, it may be the case that people higher in prejudice show a stronger HPA-axis response after being confronted with an out-group member [29], [30]. The latter suggested causal direction would be in line with the idea that people higher in prejudice expect less successful interactions, and therefore more strongly engage the HPA axis, e.g., to benefit from the restorative functions of cortisol. Importantly, these two possible causal directions are not mutually exclusive—that is, the cortisol–prejudice link may also be bidirectional. While very speculative, this idea would imply that the HPA-axis response and expressions of prejudice are part of a ‘vicious cycle’, which might be part of the explanation for why prejudice is such a stubborn phenomenon [31]. Directly testing these possibilities is an important avenue for future research.

Our findings may be further understood in terms of fMRI (functional magnetic resonance imaging) and ERP (event-related potential) research on prejudice-related processes. In such research, expressions of prejudice are often found to be a function of basic emotional responses (mediated by the amygdala), and higher-level control processes (mediated by the medial and lateral prefrontal cortex, mPFC and lPFC; and the anterior cingulate cortex, ACC) [32], [33]. While people may have initial negative reactions towards out-group members (amygdala), higher-level control processes may subsequently curb the influence of these basic emotional responses [34], preventing them from affecting overt behavior. Such control may especially occur when these emotional responses are less relevant or when social desirability is a concern (e.g., due to interpersonal similarity [35], or due to the contemporary norm to act unprejudiced [36]). The operation of these control processes—and more specifically, the process that prevents cortisol from affecting overt self-reports—may well underlie the finding that the cortisol–prejudice relation was absent for anticipated interactions with out-group members similar to the self. This explanation converges with the idea that the ACC and the PFC are not only central to the regulation of prejudicial impulses [32], but that they also serve to regulate HPA-axis activity in several ways [37].

While the current work employed an experimental procedure commonly used in social psychology to simulate the anticipation of an intergroup interaction, an important question for future research is how cortisol responses that occur to other inter-group related events (e.g., mere exposure to out-group members, exposure to inter-group conflicts, actual interactions that have positive vs. negative outcomes) affect prejudice. Such research would potentially be fruitful, as it would help to paint a more generalizable picture of how the HPA axis affects prejudice. Still, the current findings have important implications for the study of prejudice, and they may lead to the generation of new predictions. For example, HPA-axis reactivity is known to be a function of age, sex, and many other demographic, physical, situational, and personality characteristics (e.g., attachment style) [38]–[42]. It would be interesting to see whether such factors also play a role in the occurrence—and perhaps also the development—of prejudicial attitudes. Furthermore, the administration of dietary supplements (or drugs) that suppress HPA-axis activity may potentially diminish prejudicial attitudes [43]. Adopting such a biological perspective may in the future help to better understand (and perhaps also to prevent) the occurrence of prejudicial attitudes and behavior.

## Supporting Information

Data S1 **The dataset on which we based our conclusions.**
(XLSX)
